# Microdroplet-Engineered Skeletal Muscle Organoids from Primary Tissue Recapitulate Parental Physiology with High Reproducibility

**DOI:** 10.34133/research.0699

**Published:** 2025-05-15

**Authors:** Jiawei Li, Yiming Yang, Ziqi Yi, Yu Zhu, Haowei Yang, Baiming Chen, Peter E. Lobie, Shaohua Ma

**Affiliations:** ^1^ Tsinghua Shenzhen International Graduate School (SIGS), Tsinghua University, Shenzhen 518055, China.; ^2^ Key Laboratory of Industrial Biocatalysis, Ministry of Education, Tsinghua University, Beijing 100084, China.; ^3^ Meatoid Biotechnology Limited, Shenzhen 518107, China.; ^4^ School of Medicine, The Chinese University of Hong Kong, Shenzhen 518172, China.; ^5^ Key Lab of Active Proteins and Peptides Green Biomanufacturing of Guangdong Higher Education Institutes, Tsinghua Shenzhen International Graduate School, Shenzhen 518055, China.

## Abstract

Achieving high maturity and functionality in in vitro skeletal muscle models is essential for advancing our understanding of muscle biology, disease mechanisms, and drug discovery. However, current models struggle to fully recapitulate key features such as sarcomere structure, muscle fiber composition, and contractile function while also ensuring consistency and rapid production. Adult stem cells residing in muscle tissue are known for their powerful regenerative potential, yet tissue-derived skeletal muscle organoids have not been established. In this study, we introduce droplet-engineered skeletal muscle organoids derived from primary tissue using cascade-tubing microfluidics. These droplet-engineered organoids (DEOs) exhibit high maturity, including well-developed striated sarcomeres, spontaneous and stimulated contractions, and recapitulation of parental muscle fiber types. Notably, DEOs are produced in just 8 d without the need for primary cell culture—substantially accelerating the 50- to 60-d process required by classical organoid models. Additionally, the cascade-tubing microfluidics platform enables high-throughput production of hundreds of uniform DEO replicates from a small tissue sample, providing a scalable and reproducible solution for skeletal muscle research and drug screening.

## Introduction

Skeletal muscle serves as the primary actuator in animals. Its highly structured, hierarchical organization spans multiple assembly levels: from the sarcomere, the most fundamental contractile unit, to myofibrils, muscle fibers, fascicles, and, ultimately, the entire muscle. The precise alignment and coordination of sarcomeres, along with the orchestrated communications of multiple cell compositions, ensure the efficient and controlled contractions necessary for complex locomotion. Skeletal muscle contains multiple fiber types, varying based on species and body region. These fibers are typically classified into type I (slow-twitch), type IIA, and type IIB (fast-twitch), each contributing differently to muscle contraction, endurance, and power [1]. Skeletal muscle also functions as a major metabolic organ, accounting for nearly 80% of glucose uptake in the postprandial state [2].

Less noticed but highly impactful, skeletal muscle disorders markedly reduce life quality across all age groups [3–5]. However, many muscle disorders have no cures, e.g., Duchenne muscular dystrophy (DMD) [6,7], amyotrophic lateral sclerosis [8], sarcopenia [9], myotonic dystrophy [10], and dermatomyositis [11]. Even for disorders with existing treatments, such as diabetic muscle dysfunction and rhabdomyolysis, many challenges remain unresolved. Although the relationship between diabetes and muscle loss has been extensively studied [4], no direct muscle-targeted therapeutic drugs have been developed to specifically preserve or restore diabetic muscle function. Similarly, while clinical treatments for rhabdomyolysis are available, the condition still accounts for 8% to 15% of acute renal failure cases [12], underscoring a persistent gap in effective prevention and management strategies. Moreover, a comprehensive understanding of the precise causes of rhabdomyolysis remains elusive [13], limiting the development of more targeted and preventive interventions. In addition, although 2-dimensional induced pluripotent stem cell (PSC)-derived myoblasts showed utility in high-throughput muscle toxicity screening [14], the development of an in vitro 3-dimensional (3D) high-throughput muscle model that preserves the native microenvironment and functionality has yet to be achieved. A key reason for the gap is the lack of reproducible and physiologically relevant models. Traditional 2-dimensional cell models face challenges in replicating the complex 3D microenvironment and gene profiling [15]. Although 3D tissue engineering approaches, e.g., Curi Bio Mantarray [16], offer improved 3D culture conditions and biomechanical cues, challenges remained in addressing phenotype recapitulation, maintaining tissue heterogeneity. Additionally, current muscle tissue engineering models predominantly rely on manual or semiautomated post-based methods, such as 2-post spindle-shaped microtissues [17,18] and 1-post ring-shaped microtissues [19,20]. These designs impose several limitations, including low throughput, typically restricting muscle tissue engineering to fewer than 100 parallel experiments. Furthermore, a substantial number of initial cells is required and compatibility with primary cells is often poor. The reliance on cell self-aggregation toward posts contributes to limited success rates. Animal models have proven efficient in addressing many scientific problems [21,22]; however, achieving accurate phenotypic recapitulation can be challenging in certain scenarios. DMD typically manifests with mild symptoms in mice, limiting their relevance for translation to human conditions [23]. These challenges highlight the urgent need for reliable, reproducible, and high-throughput in vitro models to better study muscle biology, understand disease mechanisms, and evaluate potential therapies.

Organoids, as tissue mimics and clinically relevant models, have demonstrated promising outcomes in the brain [24], liver [25], and pancreas [26]. Among the representative skeletal muscle organoid (SkMO) works (Table S1) [18,27–30], challenges persist in developing high-throughput, reproducible SkMOs that simultaneously achieve tissue recapitulation, sarcomere structure, functionality, fiber type recovery, and rapid modeling. The majority of current approaches rely on PSCs, which undergo a sequential differentiation process that includes mesodermal commitment, myogenic specification, and subsequent myogenic growth [28,29]. Although this method allows for the differentiation of multiple cell types, it is inefficient and requires lengthy differentiation times, often taking approximately 2 months [28,29]. In contrast, SkMOs derived from adult stem cells (ASCs) offer a more rapid differentiation process; however, the initial low yield of primary muscle stem cells necessitates an extended primary expansion period [30]. The extended modeling times make them unsuitable for clinical applications. Additionally, the manual handling and uncontrolled self-aggregation processes contribute to great challenges in consistency and throughput. Collectively, developing a reproducible high-throughput primary SkMO model that simultaneously meets clinical requirements—gene and protein recapitulation, fiber type recovery, rapid maturation within 10 d, contraction frequency response, and high functional maturity—remains an unsolved challenge.

To address the shortage of reproducible high-throughput muscle clinical models, we developed functional mouse skeletal muscle droplet-engineered organoids (DEOs) from primary tissue using cascade-tubing microfluidics (CTM), with nonengineered organoids (NEOs) manually pipetted as controls. CTM utilizes microfluidic tubing instead of photolithography chips for experimental purposes [31–33]. In CTM-based microdroplet generation, the oil phase and aqueous phase converge at a T-junction, resulting in the formation of monodisperse microdroplets. Compared to lithography chips, CTM enables surfactant-free microdroplet generation while maintaining a sequential, ordered queue. DEOs exhibited several key features: (a) high maturity and functionality: Striated sarcomere structures were well recovered, and DEOs recapitulated parental muscle fiber types both genetically and phenotypically. Spontaneous and stimulated contractions were robustly observed, including frequency response, wave summation, and tetanic feature recapitulation. The use of DEOs as a model for muscle toxicity and disease was also investigated. (b) Rapid modeling time: Without the need for primary culture, SkMOs were directly developed from tissue-isolated primary cells in just 8 d. By harnessing the potential of primary ASCs, DEOs matured into contractile SkMOs via an accelerated, 2-step serum-free culture process, substantially enhancing their potential for clinical translation. (c) Consistent, high-throughput production: The DEO platform enabled the automated generation of hundreds of uniform SkMO replicates from a small piece of tissue, ensuring reproducibility and scalability.

## Results

### Development of SkMOs

**Fig. 1.**
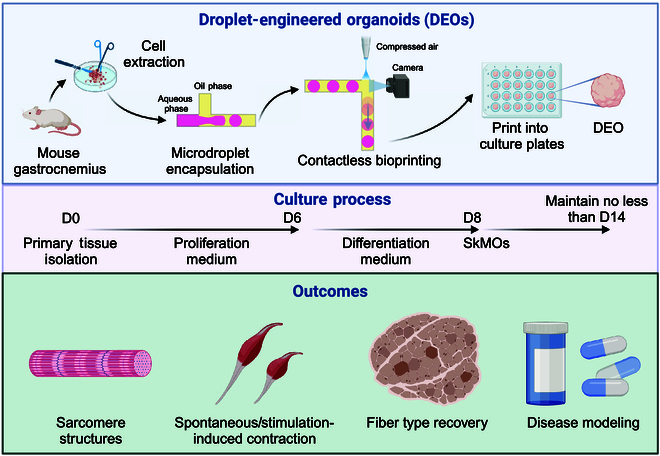
Schematic diagram of the production and outcome of skeletal muscle organoids (SkMOs).

**Fig. 2.**
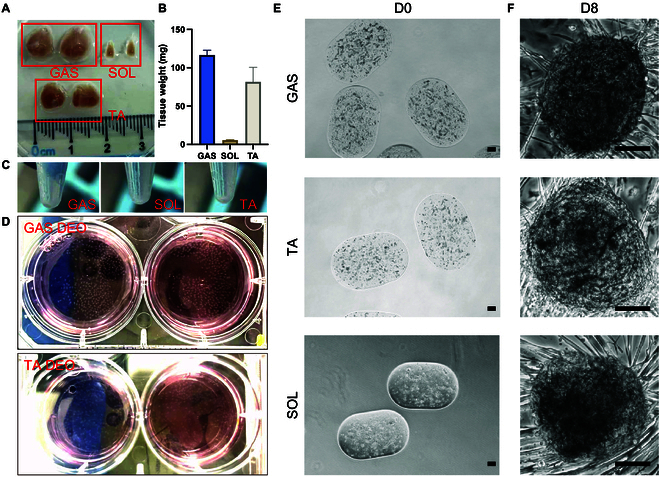
Generation of droplet-engineered organoids (DEOs) from gastrocnemius (GAS), tibialis anterior (TA), and soleus (SOL). (A) Isolation of GAS, SOL, and TA muscles from a single mouse. (B) Muscle weight comparison of GAS, SOL, and TA in (A). (C) Cell pellets obtained after tissue digestion, red blood cell lysis, and cell straining from the samples in (A). (D) Generation of GAS and TA DEOs from the isolated muscle cells. (E and F) Bright-field images of GAS, TA, and SOL DEOs at D0 (E) and D8 (F), showing their initial formation and subsequent development. Scale bars: 100 μm.

Primary SkMOs were generated using 2 distinct methodologies: nonengineered manual pipetting NEOs and engineered CTM-based DEOs (Fig. 1). In both approaches, the process commenced with mechanical and enzymatic digestion of gastrocnemius muscle tissue, followed by embedding primary skeletal muscle cells in Matrigel, a commonly used extracellular matrix (ECM). NEOs were produced by manually pipetting the cell-laden Matrigel into 10-μl domes, while DEOs were formed using the CTM system to generate ~0.1 μl (~500 μm in diameter) of microdroplet organoid precursors, which were subsequently 3D-printed into culture plates. The unidiameter polytetrafluoroethylene (PTFE) tubing provided a continuous flow path without abrupt changes in the flow field. Following droplet formation at the T-junction, droplets were temporarily stored in the long PTFE tubing, remaining in sequence without mixing. This methodological distinction permitted the DEO to achieve a markedly larger production scale, a more uniform organoid size, and a consistent cell distribution. The gastrocnemius muscle isolated from a single male mouse can typically yield over 500 DEOs (Fig. 2A to D) without requiring primary cell expansion, providing enough organoids to load five 96-well plates for drug screening. The droplet-engineered method is independent of the type of parental muscle types (Fig. 2E and F) or species, consistent with our previous research [31–35]. This indicates that the method can be seamlessly adapted for generating human muscle organoids as well. The spherical droplet template of DEOs was employed to mitigate any potential geometrically induced discrepancies in intercellular communication. Given the pronounced influence of intercellular distance on intercellular communication, which is typically effective up to approximately 250 μm [36], the maximum distance from the DEO center to any cell was maintained at around 250 μm. In contrast, the maximum distance from the NEO center to its outer boundary exceeded 2 mm, resulting in a less uniform intercellular communication environment in NEOs.

**Fig. 3.**
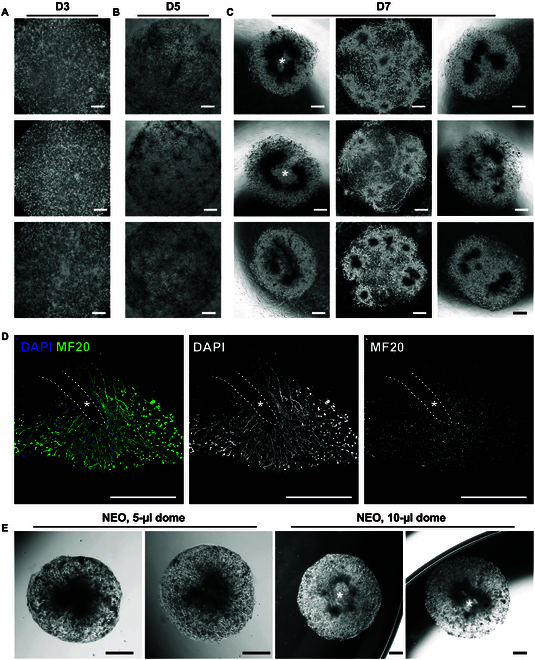
Nonengineered organoid (NEO) spatial pattern and development. (A to C) Bright-field images of NEOs at different time points (D3, D5, and D7). (C) By D7, various developmental patterns were observed, including ring-shaped structures with a low-cell-density central zone (left column, asterisk indicating the center), multicolony morphology with myofiber connections (middle column), and other structural variations (right column). (D) Immunofluorescence staining of MF20 in NEOs revealed a spatial distribution pattern, with 4′,6-diamidino-2-phenylindole (DAPI) counterstaining marking nuclei. (E) Effect of gel volume (5 μl vs. 10 μl) on NEO development, where 5-μl constructs exhibited a radial morphology, while 10-μl constructs developed a central low-cell-density zone. Scale bars: 500 μm.

NEO development consistency and pattern was first investigated. NEOs exhibited a strong spatial development pattern. Although initially forming from a uniform cell density and distribution (Fig. 3A), by D5, cells aggregated into multiple small colonies, with intercolony connections emerging (Fig. 3B). This observation suggests that cells preferentially integrate within close proximity, leading to NEOs developing from multiple sub-colonies rather than as a single homogeneous structure. By D7, distinct NEO developmental patterns emerged (Fig. 3C). The first pattern was a ring-shaped structure characterized by a sparse–dense–sparse cell distribution (Fig. 3C, left column). The second pattern consisted of multiple colonies connected by intercolony myofibers (Fig. 3C, middle column). Additional variations in morphology were also observed (Fig. 3C, right column). To further validate these spatial patterns, we performed whole-mount MF20 immunofluorescence staining of NEOs. The myofibers exhibited a central-to-peripheral orientation, with a low-cell-density central zone (Fig. 3D, the asterisk indicating the center, and the dashed line marking the boundary). This correlated with bright-field observations, reinforcing the structural organization of NEOs. The distance between the low-cell-density zone boundary and the NEO gel boundary was approximately 500 μm, which may partly explain why DEOs exhibited more consistent outcomes. Next, we examined the effect of the initial gel volume on NEO morphology. We hypothesized that geometry-induced nutrient supply inconsistencies contributed to the spatial organization and ring-shaped structure formation of muscle NEOs, as we proved in nutrient-supply-affected lung organoid development in previous research [33]. If this hypothesis were correct, modifying the initial gel volume should alter the size and distribution of the ring-shaped pattern. We compared NEOs developed from 5- and 10-μl gels (Fig. 3E); 5-μl NEOs were smaller in gel size and exhibited a high cell density in the center with lower density at the periphery, lacking a central low-cell-density zone. Radially oriented myofibers were observed. In contrast, 10-μl NEOs displayed a ring-shaped morphology with a distinct central low-cell-density zone, supporting the hypothesis that geometry influences spatial patterning. Collectively, these results suggest that primary skeletal muscle cells develop through close-proximity interactions, rather than forming a uniform structure. Geometric constraints greatly impact organoid consistency, differentiation, and developmental patterning, emphasizing the role of spatial organization in skeletal muscle model optimization. This insight prompted us to further explore the comparison between NEOs and DEOs.

**Fig. 4.**
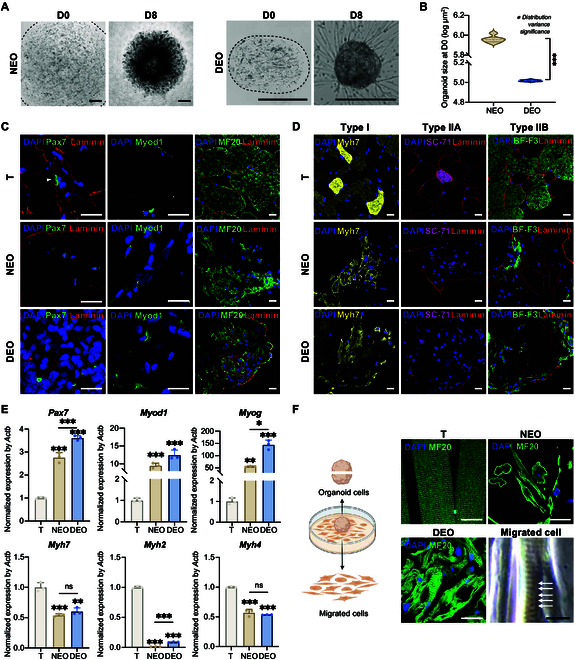
SkMOs recapitulate parental tissue development. (A) Bright-field images of a NEO and a DEO at D0 and D8. The black dashed line represents the boundaries of the NEO and DEO. Scale bars: 500 μm. (B) Violin plot of organoid size at D0 on a log scale. Distribution variance was assessed using Levene’s test to compare differences in variability. (C) Immunofluorescence staining of skeletal muscle differentiation markers, including Pax7, Myod1, and MF20, counterstained with laminin and DAPI. Scale bars: 20 μm. (D) Immunofluorescence staining of the skeletal muscle fiber type markers Myh7, SC-71, and BF-F3 for type I fibers (Myh7), type IIA fibers (Myh2), and type IIB fibers (Myh4), respectively, counterstained with laminin and DAPI. Scale bars: 20 μm. (E) Reverse transcription quantitative polymerase chain reaction (RT-qPCR) analysis of the gene expression of *Pax7*, *Myod1*, *Myog*, *Myh7*, *Myh2*, and *Myh4*. Statistical analysis was performed using one-way analysis of variance (ANOVA), with results presented as mean ± standard deviation (SD). (F) Immunofluorescence analysis of sarcomere formation in parental tissue (T), a NEO, and a DEO along with bright-field images of sarcomeres from emigrated DEO cells. The striated morphology and alternating bright and dark bands indicate successful sarcomere organization. Scale bars: 20 μm. Statistical significance is denoted as follows: * for *P* < 0.05, ** for *P* < 0.01, and *** for *P* < 0.001.

The development of SkMOs notably reduced the culture period to 8 d and simplified the overall procedure by 2-step development without any primary culture. Following primary tissue isolation on D0, organoid precursors were cultured in a skeletal muscle proliferation medium for 6 d, and then they were switched to a differentiation medium for 2 d to become mature SkMO at D8. The organoids can be maintained in the differentiation medium for at least another 6 d. Mature SkMOs exhibited a highly ordered sarcomere structure, demonstrating functional contraction and the recovery of parental tissue muscle fiber types. As illustrated in Fig. 4A, NEOs and DEOs demonstrated dome- and elliptical-shaped morphologies at D0, respectively, with an identical initial cell density. To assess reproducibility, organoid size was quantified at D0 (Fig. 4B). This revealed that DEOs exhibited a significantly narrower size distribution compared to NEOs. The increased variability observed in NEOs is likely due to the manual pipetting process, which introduces inconsistencies in dome size and structural integrity. This observation highlights the advantage of DEOs in achieving more consistent structural uniformity, which is essential for reproducible functional outcomes.

Both organoids underwent a reduction in size during the differentiation process, which is likely driven by cellular self-organization, intercellular connection formation, and the generation of contractile forces. DEOs demonstrated adherence to the culture plate and exhibited cell migration throughout the culture process. By D8, DEOs exhibited a more uniform and dense cell distribution compared to the light–dense–light pattern observed in NEOs. Both NEOs and DEOs exhibited spontaneous contraction by D8 (Movies S1 and S2). DEOs can be cultured using either tissue-treated plates or ultralow-attachment plates, offering flexibility based on application requirements. Both attached and suspension cultures successfully produce contractile DEOs. On tissue-treated plates, DEOs typically proliferate for 3 to 4 d before attaching to the plate by gravity. Consistent with recent SkMO research [30], DEOs can be considered a reservoir of myogenic progenitor (MP) cells. Cells emigrating out of a DEO are capable of proliferating, fusing into multinucleated cells, and contracting. By detaching the attached DEO from the plate through pipetting and transferring it to a new well, the DEO can reattach and continue to release migratory cells. This process can be repeated multiple times, effectively serving as a passaging method.

### Myogenic differentiation and muscle fiber type recovery

The differentiation of skeletal muscle cells within the organoids was evaluated through the examination of the expression and spatial distribution of key myogenic markers, employing both immunofluorescence and reverse transcription quantitative polymerase chain reaction (RT-qPCR) techniques. Pax7, a marker for muscle satellite cells, was expressed in the tissue (T), NEOs, and DEOs, indicating the preservation of muscle stem cells within these organoids (Fig. 4C). Additionally, Myod1, a marker of myogenic differentiation, was observed in both organoids, with a notably higher expression in DEOs. This suggests that DEOs may have a greater capacity to promote early myogenic differentiation compared to NEOs. Immunofluorescence analysis further confirmed the development of mature muscle fibers in both NEOs and DEOs, as indicated by positive MF20 staining, which recognizes all isoforms of myosin heavy chain. Laminin, a marker of the basal lamina, was observed surrounding the MF20+ regions in DEOs. However, neither organoid exhibited the well-organized laminin structure or the peripheral nuclear positioning observed in the tissue controls, where nuclei migrate from the center of the myotube to its periphery during maturation [37]. This lack of organization may be attributed to the absence of uniaxial biomechanical cues during development.

To investigate the recapitulation of muscle fiber types in SkMOs, myosin isoforms were analyzed using specific antibodies (Fig. 4D). The native gastrocnemius tissue was composed of type I, IIA, and IIB muscle fibers, as confirmed by immunofluorescence. The Myh7 marker, which identifies type I muscle fibers, exhibited robust expression in the tissue, with bright and moderately distributed Myh7+ fibers. Myh4, a marker of type IIB fibers, was the most prevalent isoform observed in the gastrocnemius muscle. In contrast, Myh2, the marker for type IIA fibers, exhibited the lowest expression levels, which is consistent with its low abundance in this muscle type. Both NEOs and DEOs were successful in recapitulating the expression patterns of these myosin isoforms. It is noteworthy that, given the loss of proliferative capacity in mature muscle fibers and the fact that a 40-μm strainer permits only single cells and small cell clusters, it is very likely that SkMOs develop primarily from the stem cell populations within the isolated cells. These stem cells somewhat retained the ability to differentiate into various muscle fiber types. We hypothesize that nonstem cells may also play a critical role in guiding differentiation within the organoids, although this hypothesis requires further validation in future studies.

Gene expression analysis by qPCR corroborated these observations (Fig. 4E), demonstrating that *Pax7*, *Myod1*, and *Myog* (a marker for myocyte fusion) were significantly upregulated in both DEOs and NEOs in comparison to those in tissue (T). Of particular note, DEOs exhibited an even higher expression of these genes than NEOs. The elevated levels of *Pax7* in DEOs indicate an enhanced capacity for maintaining satellite cell populations, which are essential for muscle regeneration. Furthermore, the markedly elevated expression of *Myog* in DEOs substantiates their superior differentiation capacity in comparison to that of NEOs. These results indicate that in vitro SkMOs followed a regenerative trajectory similar to that observed in muscle repair processes, rather than reflecting the more quiescent, stable state of mature muscle tissue. This pattern aligns with observations in organoid models of other tissues, such as the liver, where liver organoids tend to mirror the gene expression profiles seen during liver regeneration (e.g., after partial hepatectomy), rather than those of fully quiescent liver tissue [38]. This underscores the regenerative nature of organoid formation, whereby in vitro self-organization more closely resembles a wound healing or repair response. With regard to structural protein genes, DEOs and NEOs were able to successfully recapitulate *Myh7* and *Myh4* expression. However, both exhibited approximately half the expression levels observed in T. This may be attributed to the absence of further biomechanical regulation within the culture system. *Myh2* expression remained low in both organoids, which is probably because of the low abundance in the parental tissue. However, DEOs displayed significantly higher *Myh2* expression than NEOs, suggesting that DEOs may facilitate the development of type IIA fibers to a greater extent than NEOs, although at lower levels than those in native tissue.

The volume of NEOs was approximately 100 times greater than that of DEOs. To investigate spatial consistency, the MF20 and the Myh7 immunofluorescence of NEOs and DEOs were quantified by dividing images into equal-sized subimages and analyzing the distribution of marker expression (Fig. S1A). Notable heterogeneity was observed in the NEO, with clustered expression of MF20 and Myh7 confined to specific regions of the organoid. In contrast, the distribution of these markers across the entire DEO was significantly more uniform (Fig. S1B). Heatmap visualization of the ratio of the positive area to the total subimage area further emphasized the spatial inconsistency in NEOs, where the majority of regions exhibited low-intensity staining (Fig. S1C). The clustering of myosin expression in a NEO presents a critical challenge for functional applications, as it limits the organoid’s ability to mimic the uniform distribution of muscle fibers observed in native tissue. In contrast, a DEO, with its more homogeneous expression pattern, offers a more reliable model for studying muscle tissue organization and function in vitro.

The sarcomere represents the fundamental contractile unit of skeletal muscle [27]. It is one of the most important criteria of SkMOs’ successful establishment. In this study, clear striated morphology was observed in the NEO group, DEO group, and cells that emigrated from a DEO (Fig. 4F). The sarcomeres exhibited a striated morphology, similar to that observed in the parental tissue. Furthermore, cells that emigrated from the DEO during organoid development differentiated into mature muscle fibers with contractile ability (Movie S3). Under bright-field microscopy, distinct bright–dark stripes were visible during contraction, indicating proper sarcomere alignment. These results suggest that the DEO supports robust muscle maturation and functional recovery of skeletal muscle.

### Regenerative gene expression and functional recapitulation in DEOs

**Fig. 5.**
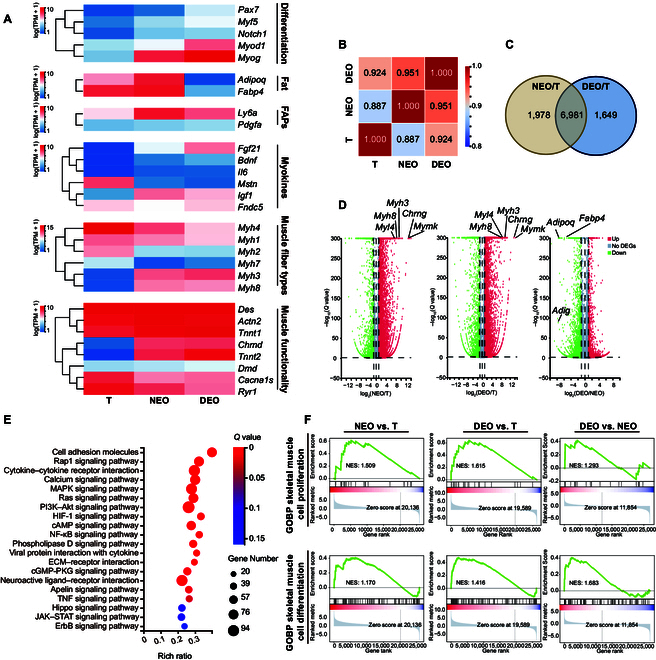
RNA sequencing (RNA-seq) analysis of T, NEOs, and DEOs. (A) Heatmap of skeletal-muscle-related genes. TPM, transcripts per million; FAPs, fibro-adipogenic progenitors. (B) Pearson correlation map of T, NEOs, and DEOs. (C) Venn diagram of the differentially expressed genes of NEO/T and DEO/T. (D) Volcano diagram of NEO/T, DEO/T, and DEO/NEO. DEGs, differentially expressed genes. (E) Kyoto Encyclopedia of Genes and Genomes pathway enrichment of DEGs of DEOs/NEOs. ECM, extracellular matrix. (F) Gene set enrichment analysis (GSEA) of the Gene Ontology (GO) biological processes of skeletal muscle cell proliferation (GO: 0014856) and skeletal muscle cell differentiation (GO: 0035914). GOBP, Gene Ontology Biological Process; NES, normalized enrichment score.

RNA sequencing (RNA-seq) was performed to compare the gene expression of T, NEOs, and DEOs. In the analysis of differentiation-related genes (Fig. 5A), SkMOs successfully mirrored the gene expression patterns characteristic of muscle regeneration. Consistent with qPCR results, DEOs exhibited an upregulated regeneration profile with higher levels of *Pax7* compared to both NEOs and native tissue, indicating a superior capacity for maintaining muscle satellite cells in vitro. Likewise, *Myod1*, *Myf5* and *Myog*, which are critical markers of myogenic differentiation, were expressed at higher levels in DEOs than in both tissue and NEOs, further reinforcing the enhanced differentiation potential observed in DEOs. The RNA-seq results for muscle-fiber-type-related genes were consistent with both the gastrocnemius muscle fiber composition and qPCR data. Among the selected gene set, *Myh4* emerged as the most highly expressed gene in the tissue samples, aligning with the fact that the mouse gastrocnemius muscle predominantly consists of *Myh4*+ type IIB fibers. Although *Myh4* expression was lower in both NEOs and DEOs compared to that in tissue, DEOs showed higher *Myh4* expression than NEOs. Similarly, *Myh2* and *Myh7* messenger RNA (mRNA) expression levels were lower in both NEOs and DEOs relative to that in tissue, although DEOs exhibited higher *Myh7* expression compared to NEOs. Both NEOs and DEOs showed upregulated neonatal muscle fiber markers *Myh3* and *Myh8*, which is consistent with the regenerative process. DEOs also demonstrated a favorable myokine expression profile, with the lowest levels of the suppressive myokine myostatin (*Mstn*) and increased levels of the supportive myokines *Fgf21* and *Igf1*, indicating a positive regulatory environment for muscle growth and regeneration. SkMOs also showed strong recapitulation of functionality genes, including desmin (*Des*), dystrophin (*Dmd*), troponin T type 1 (*Tnnt1*), nicotinic acetylcholine (Ach) receptor (*Chrnd*), and sarcomere-related alpha-actinin 2 (*Actn2*). These results indicate that the SkMOs demonstrate a high level of maturity and functionality. DEOs outperformed NEOs in parental tissue recapitulation by higher correlation with T (Fig. 5B).

DEOs exhibited upregulation of genes associated with cell–cell communication (Fig. S2). Key cell-adhesion- and junction-related genes, such as *Cdh2* (N-cadherin) and *Cdh15* (M-cadherin), which are essential for myoblast fusion and intercellular connectivity, were upregulated. Additionally, *Jup* (junction plakoglobin), a critical component of adherens junctions and desmosomes, showed increased expression, suggesting reinforced structural stability. For cell–ECM interactions, *Itga1* (integrin α1), which mediates cell anchorage to the ECM, was elevated. Similarly, *Ilk* (integrin-linked kinase), a key regulator of integrin–ECM signaling, and *Fermt2* (kindlin-2), which promotes integrin clustering and focal adhesion assembly, were upregulated, indicating enhanced ECM adhesion and mechanotransduction. Furthermore, DEOs displayed increased expression of growth factors and signaling pathway components, including *Fgf2* (fibroblast growth factor 2) and *Notch1*/*Notch3*, both of which are crucial for muscle cell proliferation, differentiation, and regeneration. Collectively, these findings suggest that DEOs establish stronger intercellular communication, contributing to superior structural organization and functional maturation compared to NEOs.

The top differentially expressed genes in both NEO/T and DEO/T comparisons were further analyzed (Fig. 5C). Both groups displayed upregulation of genes related to nascent myofibers (Fig. 5D), including *Myh3*, *Myh8*, and *Myl4*; functionality gene *Chrng* (nicotinic Ach receptor gamma subunit); and differentiation gene *Mymk* (myoblast fusion factor). The log_2_ fold change (log_2_FC) values for these genes in NEO/T were 8.92, 8.24, 7.09, 13.26, and 13.34, respectively, whereas in DEO/T, the values were 9.69, 8.05, 8.57, 13.61, and 14.31, respectively. The upregulation of *Myh3*, *Myh8*, and *Myl4* reflects the formation of nascent myofibers within the organoids, whereas the increased expression of *Mymk* suggests an active myogenesis process. The upregulation of *Chrng* is consistent with the development of functional SkMOs. Interestingly, in the comparison between DEOs and NEOs, DEOs exhibited markedly lower expression of adipose-related genes compared to NEOs, with *Adipoq*, *Fabp4*, and *Adig* showing log_2_FC values of −6.50, −5.30, and −7.77, respectively.

Kyoto Encyclopedia of Genes and Genomes enrichment on skeletal-muscle-related pathways revealed that the differentially expressed genes of DEOs and NEOs are enriched in cell-adhesion molecules, cytokine–cytokine receptor interactions, and the calcium signaling pathway (Fig. 5E). Geometrical confinement of the DEO microfluidics droplet template and microenvironment intercellular connection may contribute to this difference. The gene set enrichment analysis (Fig. 5F) of tissue, NEOs, and DEOs showed that NEOs and DEOs improved on skeletal muscle cell proliferation and differentiation, while DEOs outperform NEOs on these 2 Gene Ontology biological processes.

### Suppressed fibroblast activation in DEOs compared to that in NEOs

**Fig. 6.**
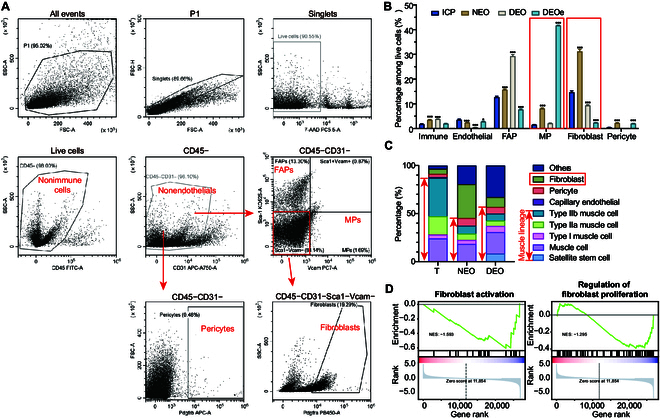
Cellular composition and fibroblast activation analysis. (A) Gating strategy of fluorescence-activated cell sorting (FACS). MP, myogenic progenitors. (B) Composition of mononuclear cells in the initial cell pool (ICP), NEOs, DEOs, and DEO-emigrated cells (DEOes). (C) Bulk RNA-seq deconvolution. Bulk RNA-seq results were mapped to the Tabula Muris Senis single-cell RNA-seq dataset [40] to calculate the cellular compositions using previously established cell deconvolution methods [50]. Arrows indicate the muscle lineage cells. (D) GSEA of fibroblast activation (GO: 0072537) and regulation of fibroblast proliferation (GO: 0048145) of DEO/NEO. Data are represented as mean ± SD. One-way ANOVA was implemented to calculate data significance. *P < 0.05; **P < 0.01; ***P < 0.001.

Except for geometrical differences, we also wondered about the biological difference in the mechanism of DEO outperformance over NEOs on tissue recapitulation and maturation. The cell composition of T, the initial cell pool, NEOs, DEOs, and DEO-emigrated cells was firstly investigated (Fig. S3). Fluorescence-activated cell sorting (FACS) adapted from a previously established protocol [39] was implemented. Cell identities were determined based on cell-specific markers (Table S2) and gating strategies (Fig. 6A). Quantification of cell compositions (Fig. 6B) revealed that fibroblasts constituted the largest population in NEOs. In contrast, DEOs and DEO-emigrated cells showed a significantly lower fibroblast percentage compared to T and NEOs. Among MPs, DEO-emigrated cells exhibited the largest MP population, followed by NEOs and DEOs. This result aligned with the observation of myofiber interorganoid connections facilitated by emigrated cells (Fig. S5). For fibro-adipogenic progenitors, DEOs had the highest fibro-adipogenic progenitor percentage, followed by NEOs, T, and DEO-emigrated cells. Regarding immune cells, both NEOs and DEOs displayed a slight increase in immune cell populations. In the endothelial cell population, all groups showed a decrease compared to T. In the pericyte population, NEOs and DEO-emigrated cells exhibited a slight increase compared to T and DEOs.

To further characterize the cell identity spectrum, bulk RNA-seq deconvolution was performed to assess cell composition at the gene expression level (Fig. 6C). Briefly, bulk RNA-seq data were mapped to single-cell RNA-seq reference datasets [40] using cellular deconvolution analysis [41]. The results were consistent with the FACS findings, showing that NEOs exhibited a higher fibroblast composition compared to DEOs and T. DEOs showed higher muscle lineage cells than NEOs. In addition, gene set enrichment analysis of bulk RNA-seq showed that DEOs had less fibroblast activation and regulation of proliferation (Fig. 6D). Collectively, NEOs exhibited enhanced fibroblast activation and overproliferation.

### Electrical stimulation and functional analysis of SkMOs

The functionality of SkMOs was assessed in response to electrical stimulation. Bright-field videos were recorded to quantify contraction amplitude using the MUSCLEMOTION algorithm [24,42,43]. For this purpose, a specialized electrical stimulation plate (ES plate; Fig. S5A) was designed to allow real-time multichannel stimulation and observation of SkMOs. The graphite component of the electrode complex in the ES plate ensured that the system did not have detrimental effects on live cells. The complete setup included an electrical pulse generator, an ES plate, and connective wires (Fig. S5B). SkMOs were seeded into the ES plate and cultured until maturation for functional analysis.

To investigate the effect of input voltage on muscle contraction, DEOs were applied at 5, 10, 15, and 20 V (Fig. S5C). No statistically significant differences were observed in the peak contraction amplitudes across the voltage levels (Fig. S5D), indicating that stimulation voltage had no measurable impact on contraction amplitude. This is likely because all available muscle fibers were recruited once the stimulation threshold was reached. Additionally, the effect of pulse duty cycle on peak morphology was examined (Fig. S5E and F). At a 5% duty cycle, sharp peaks were observed, while a double-peak morphology emerged at 10%, with the first peak showing a higher amplitude. As the duty cycle increased beyond 15%, the 2-peak pattern became more pronounced, with the higher peak shifting from the first to the second. This phenomenon is likely due to secondary contractions occurring during continuous stimulation at higher duty cycles. Therefore, pulses with an amplitude of 10 V and a duty cycle of 5% were implemented for the purpose of examining the function of the SkMOs.

**Fig. 7.**
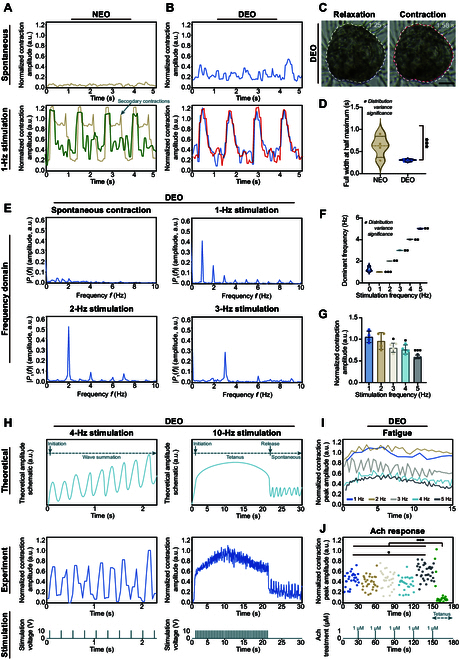
Electrical stimulation and functional analysis of SkMOs. (A) Normalized contraction amplitude of NEO during spontaneous contraction (top) and 1-Hz electrical stimulation (bottom). The green line indicates a repeat experiment. The amplitudes were normalized to the peak amplitude of NEOs under 0.5-Hz stimulation. (B) Normalized contraction amplitude of DEOs during spontaneous contraction and 1-Hz electrical stimulation. The red line indicates a repeat experiment. The amplitudes were normalized to the peak amplitude of DEOs under 0.5-Hz stimulation. (C) Bright-field images of a DEO in relaxation (1.25 s) and contraction (1.58 s) phases (Movie S5). The white and red dashed lines indicate the boundaries of the DEO during the relaxation and contraction phases, respectively. Scale bar: 100 μm. (D) Full width at half maximum (FWHM) comparison of the contraction peaks of a NEO and a DEO. Statistical analysis was performed using Levene’s test. (E) Fast Fourier transform (FFT) of DEO contraction during spontaneous contraction and 1-, 2-, and 3-Hz electrically induced contractions. The magnitude of a frequency component is represented by *P*_1_(*f*). The contraction amplitudes in the frequency domain were normalized to the amplitude at the dominant frequency under 0.5-Hz stimulation. (F) A violin plot showing the distribution of dominant frequencies from repeat DEO experiments under spontaneous contraction and 1-, 2-, 3-, 4-, and 5-Hz electrical stimulation. The distribution variance was evaluated using Levene’s test. (G) Normalized contraction amplitude of DEOs at 1-, 2-, 3-, 4-, and 5-Hz electrical stimulations. Contraction amplitudes were normalized to the average contraction amplitude at 1-Hz stimulation. (H) Wave summation and tetanic contraction of DEOs. The top panel presents a theoretical illustration of wave summation and tetanic contraction patterns. The middle panel depicts the experimental DEO contraction amplitudes, which were normalized to the contraction amplitude observed under 0.5-Hz electrical stimulation. The bottom panel displays the stimulation pattern, with each vertical line representing a 10-V pulse. (I) The fatigue properties of DEOs under different frequency stimulations. Normalized peak contraction amplitudes are shown for 1-, 2-, 3-, 4-, and 5-Hz stimulations. (J) A scatter plot demonstrating the normalized peak amplitudes of DEOs in response to increasing concentrations of acetylcholine (Ach). Ach was added to the culture medium in a sequential manner, achieving final concentrations of 1, 2, 3, 4, and 5 μM. The peak amplitudes were normalized to the maximum peak amplitude. Statistical analysis was performed using one-way ANOVA, with data presented as mean ± SD. Statistical significance is indicated as *P < 0.05, **P < 0.01, and ***P < 0.001.

In comparison to the low-amplitude spontaneous contractions, both NEOs and DEOs exhibited periodic contractions in response to electrical stimulation (Fig. 7A and B and Movies S4 and S5). The contraction and relaxation phases were clearly visible under the microscope (Fig. 7C). However, NEOs and DEOs displayed distinct contraction patterns. NEOs exhibited both sharp peak contractions and double-peak contractions in different repeat experiments. In the double-peak pattern, partial relaxation was observed before the secondary contraction was completed. In contrast, DEOs consistently displayed sharp peak contractions across all experiments (Fig. 7B). The discrepancy in contraction patterns can be attributed to the suboptimal maturation level of NEOs compared to that of DEOs. To quantify the contraction pattern difference, the full widths at half maximum of NEO and DEO contraction peaks were compared. The results indicated that NEOs exhibited significantly greater variability and a notably larger mean full width at half maximum value than DEOs, suggesting that DEOs consistently displayed a sharp peak morphology (Fig. 7D). Overall, DEOs demonstrated more consistent size, spatial expression, and contraction patterns compared to NEOs. Therefore, DEOs were selected for subsequent functional analyses.

A Fourier transformation was performed on the time-domain DEO contraction amplitude to examine the frequency characteristics of the contraction (Fig. 7E). The frequency spectrum of the spontaneous contraction displayed a flat profile, indicating the occurrence of multiple spontaneous contraction frequencies. The electrical stimulation groups exhibited discernible peaks at the corresponding stimulation frequency, in addition to minor peaks at multiple frequency locations (Fig. 7E). For instance, the 2-Hz stimulation-induced contraction displayed a markedly pronounced peak, indicating that the DEO contraction adheres closely to the stimulation frequency. Moreover, the dominant frequency of the repetitive experiments exhibited a markedly narrower distribution than that of the spontaneous contraction (Fig. 7F). The DEO demonstrated robust functionality in responding to external electrical stimulations. As the stimulation frequency increased, the peak amplitude exhibited a gradual decline (Fig. 7G), which may be attributed to the incomplete relaxation of the SkMO prior to the arrival of the subsequent stimulation pulse.

Wave summation and tetanic contraction are key functionality characteristics of skeletal muscle. Temporal summation occurs when successive stimuli are applied in rapid succession, resulting in the force of each contraction building on the previous one before the muscle fully relaxes. This leads to a stronger overall contraction. Tetanus refers to a sustained muscle contraction resulting from a rapid series of stimuli that prevent relaxation between contractions, thereby leading to a continuous and forceful contraction. At a frequency of 4 Hz, the normalized contraction peak amplitude of a DEO exhibited an increase over time, indicative of the occurrence of temporal summation (Fig. 7H). At 10-Hz stimulation, the occurrence of tetanic contraction was observed (Movie S6). Upon the commencement of electrical stimulation, the DEO exhibited a rapid transition from spontaneous contractions to large-amplitude contractions, accompanied by minor fibrillation. Upon cessation of high-frequency stimulation, the DEO exhibited a return to its baseline state, accompanied by a resumption of spontaneous contractions. It is noteworthy that the amplitude of these spontaneous contractions after 10-Hz stimulation was greater than that observed prior to stimulation. This increase may be attributed to elevated intracellular calcium concentrations, which may enhance contraction even after stimulation has ceased. The wave summation and tetanic contraction behavior of a DEO closely resembles that observed in real skeletal muscle, indicating that skeletal muscle functionality has been successfully recapitulated in a DEO.

Fatigue is one of the inherent characteristics of skeletal muscle. Over time, the peak contraction amplitude gradually decreased with continued stimulation, a trend consistently observed across stimulation frequencies from 1 to 5 Hz, indicating that a DEO undergoes contraction fatigue over time, similar to natural muscle (Fig. 7I).

To further assess the functionality of DEOs, the reaction to the neuromuscular transmitter Ach was investigated. Ach is an excitatory neurotransmitter that induces contraction in normal skeletal muscle upon stimulation. To evaluate the DEO response to Ach, we proceeded to sequentially add Ach (1 μM each time) to the culture medium and recorded the contractions until a stable pattern was achieved (Movie S7). No notable alteration in peak amplitude was discerned with Ach concentrations ranging from 0 to 3 μM (Fig. 7J). However, at 4 μM Ach, a significant increase in peak amplitude was observed, indicating a stronger contraction. Upon increasing the Ach concentration to 5 μM, an immediate strong contraction was observed, which then transitioned into a tetanic contraction, characterized by significantly lower peak amplitudes. While the exact Ach concentration at which the DEO transitioned from a nonsignificant change to a tetanic contraction varied among individual samples, the overall pattern remained consistent. These results demonstrate that the DEO can respond appropriately to Ach treatment, effectively recapitulating normal skeletal muscle function.

### Muscle DEOs as a pharmacological response and metabolic pathology model

**Fig. 8.**
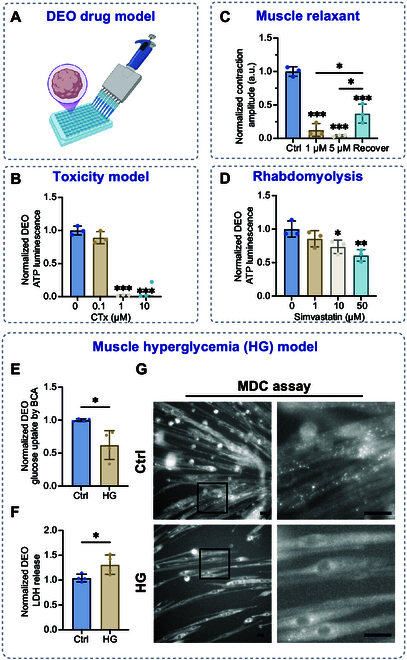
DEOs as a drug screening model for muscle toxicity and metabolism. (A) Schematic representation of DEO-based drug screening. (B) Adenosine triphosphate (ATP) assay measuring DEO viability under treatment with 0, 0.1, 1, and 10 μM cardiotoxin (CTx). (C) Normalized contraction amplitudes of DEOs under treatment with the muscle relaxant dantrolene (0, 1, and 5 μM) and after a 15-min recovery period. (D) ATP assay of DEO viability following treatment with 0, 1, 10, and 50 μM simvastatin. (E) Glucose uptake analysis in DEOs treated with control (Ctrl) and hyperglycemia (HG) conditions, quantified using 2-(*N*-(7-nitrobenz-2-oxa-1,3-diazol-4-yl)amino)-2-deoxyglucose (2-NBDG) uptake and normalized by the bicinchoninic acid (BCA) assay. (F) Lactate dehydrogenase (LDH) release assay in DEO supernatants under Ctrl and HG conditions, indicating cytotoxicity. (G) Monodansylcadaverine (MDC) staining assay of DEOes under Ctrl and HG conditions. Scale bars: 100 μm. Statistical analysis was performed using one-way ANOVA, with data presented as mean ± SD. Statistical significance is indicated as *P < 0.05, **P < 0.01, and ***P < 0.001.

To evaluate the utility of DEOs as a physiology-relevant model, a proof-of-principle experiment was performed (Fig. 8A). Initial experiments employed cardiotoxin, a myolytic peptide isolated from *Naja atra* venom, which compromises sarcolemmal stability [44]. A 24-h treatment with cardiotoxin at no less than 1 μM resulted in marked adenosine triphosphate (ATP) depletion (*P* < 0.001), demonstrating concentration-dependent cytotoxic effects (Fig. 8B). To characterize DEO pharmacodynamics, contractile responses to dantrolene—a ryanodine receptor antagonist that blocks sarcoplasmic reticulum calcium efflux [45]—were analyzed. Dantrolene exposure induced progressive attenuation of contractile amplitude, with partial inhibition at 1 μM (intermittent residual contractions) and complete cessation at 5 μM (Fig. 8C). Partial functional recovery was observed after a 15-min washout period, highlighting reversible pharmacological modulation.

Simvastatin, a statin linked to clinical rhabdomyolysis [46,47], was tested to assess DEO toxicity profiling. Acute 24-h simvastatin exposure triggered a concentration-dependent reduction in cellular viability, evidenced by diminished ATP levels (Fig. 8D). These results mirror clinical patterns of statin-associated myotoxicity, underscoring the platform’s ability to replicate drug-induced pathology.

Type 2 diabetes is marked by chronic hyperglycemia (HG) due to insulin resistance or deficient secretion [48]. Skeletal muscle, a major site of glucose metabolism, plays a critical role in type 2 diabetes progression. Insulin resistance disrupts GLUT4 membrane translocation, reducing glucose uptake and exacerbating systemic HG [49]. To investigate the effects of HG on SkMOs, we cultured DEOs under control (Ctrl) and HG conditions. Glucose uptake was quantified using the fluorescent tracer 2-(*N*-(7-nitrobenz-2-oxa-1,3-diazol-4-yl)amino)-2-deoxyglucose (2-NBDG). HG DEOs exhibited reduced 2-NBDG incorporation compared to Ctrl, mimicking the impaired glucose utilization in vivo (Fig. 8E). Lactate dehydrogenase (LDH) release was significantly elevated in HG cultures, indicating membrane disruption under stress. Monodansylcadaverine (MDC) staining confirmed autophagosome accumulation, with increased fluorescent puncta in HG-treated groups. These data demonstrate that chronic HG triggers dual pathological effects in SkMOs: enhanced autophagic activity and diminished glucose uptake capacity.

Collectively, these findings validate DEOs as a robust platform for modeling drug responses and metabolic dysfunction, bridging in vitro observations with clinical disease mechanisms.

## Discussion

Skeletal muscle is the most abundant tissue and the largest metabolic organ in the human body. However, reliable and reproducible in vitro models remain challenging. Given the challenge of obtaining sufficient primary human skeletal muscle cells from small biopsies for precision medicine, we addressed this limitation by using DEOs for scalable production under low-cell-yield conditions [33]. In this pilot study, we established functional primary mouse SkMOs as a proof-of-concept model and successfully developed contractile skeletal muscle DEOs in a high-throughput manner. This DEO platform outperforms classical skeletal muscle models in several key aspects:

• ASC-derived SkMOs with sarcomere and muscle fiber type recovery: The majority of previous organoid studies have employed PSCs, which present considerable challenges due to their prolonged culture periods, suboptimal phenotype recapitulation, and limited prospects for clinical application. We effectively utilized ASCs to rapidly establish sarcomeres and maintain type I, type IIA, and type IIB muscle fibers within the organoids. This achievement is crucial for replicating the functional characteristics of muscle in vitro and underscores the potential of DEOs for muscle disease modeling and therapeutic applications.
• Contractile functional SkMOs: One of the major advancements of the model herein is its stable contractility. Both spontaneous contractions and electrically induced patterned contractions were observed in DEOs, demonstrating the essential contractile functionality required for muscle models. Typically, contractile activity begins around day 6 and is stably maintained thereafter.
• High-throughput production in 8 d: The engineered CTM system enabled the high-throughput production of DEOs. Remarkably, gastrocnemius isolated from one mouse could generate over 500 DEOs with a consistent size and reproducible outcomes in just 8 d, without the need for primary culture. This large production capacity opens new possibilities for human-patient-derived SkMOs in drug screening. Additionally, the scalability of DEO production holds potential for a range of applications, including the assembly of larger muscle tissues by migration-based assembly (Fig. S4), biorobotics, and living materials.

In addition to these advancements, the current study contributes a streamlined fast-modeling protocol, ready-to-use electrical stimulation hardware, and a standardized evaluation workflow for SkMO research. These resources will enable other researchers to adopt and expand upon this approach, potentially accelerating progress in the field of SkMOs. The SkMO protocol enables rapid functional establishment of organoids with a high success rate, with nearly all DEOs exhibiting contractile activity by day 8. The protocol consists of only 2 stages, making it considerably simpler than existing methods. Additionally, a low-cost electrical stimulation device for SkMOs was developed, allowing for real-time monitoring of contractions. In this study, frequency response, wave summation, fatigue, and Ach response into the contraction analysis were also incorporated. Combined with gene expression, protein expression, sarcomere structure, and muscle fiber type analysis, a standardized, multimodal evaluation workflow for SkMO research was established.

Despite the promising results, this study also has several limitations. One key limitation is the lack of large-scale sarcomere alignment within the organoids. Although the formation of functional sarcomeres was successfully demonstrated, achieving uniform and widespread alignment across the entire organoid remains a challenge. Sarcomere alignment is crucial for ensuring the coordinated contractile function of skeletal muscle, and future studies will need to address this issue to further improve the model. Another limitation is the incomplete recapitulation of structural gene expression. Despite the effective expression of key markers of muscle fiber types and differentiation by DEOs, the overall expression levels of structural genes were observed to be lower than those seen in native tissue. This indicates that additional optimization of culture conditions, including biomechanical cues, may be necessary to fully replicate the intricate structural components of skeletal muscle in vitro. Moreover, although DEOs demonstrated the potential to reproduce normal muscle function, they have yet to be validated as a disease model. Testing DEOs for disease recapitulation, such as modeling diabetic muscle organoids, and evaluating drug responses, such as muscle reactions to semaglutide, will be crucial for assessing their utility in studying disease mechanisms and screening potential therapeutic interventions. Furthermore, the present study was conducted using organoids derived from mice, and future work will need to focus on adapting the system for human cells to enhance its clinical relevance.

In addition to addressing these limitations, several future research directions hold notable promise. One potential avenue is the exploration of organoid assembly, wherein multiple tissue types are integrated to form more complex systems that better mimic interorgan communications. A pilot study of DEO sparse assembly was also performed. Cells emigrated from DEOs formed contractile inter-DEO connection muscle fibers (Fig. S4). Another important area of investigation is secondary myogenesis within organoids, which would enable modeling of later stages of muscle development and regeneration. Furthermore, we aim to explore muscle fiber type transitions in SkMOs, which could offer valuable insights into the roles that different fiber types play in muscle function and the progression of muscle-related diseases.

## Materials and Methods

### Isolation of primary skeletal muscle cells from BALB/c mice

Primary skeletal muscle cells were isolated from 4- to 6-week-old male BALB/c mice, sacrificed using CO_2_ asphyxiation. The gastrocnemius muscles from both hind limbs were carefully excised, and visible connective tissue and subcutaneous fat were manually removed. The muscle tissue was finely minced into 1- to 3-mm^3^ fragments using sterile scissors and scalpels. All animal experiments were conducted in accordance with the ethical guidelines and approved by the Animal Experimentation Ethics Committee at Tsinghua Shenzhen International Graduate School. The procedures adhered strictly to national and institutional guidelines for the care and use of laboratory animals.

The minced tissue was enzymatically digested in Dulbecco’s modified Eagle medium/F12 medium (Gibco, 11330032), supplemented with 2 mg/ml collagenase I (Yuanye, S10053) and 1% penicillin–streptomycin (Gibco, 15140122), at 37 °C for 40 to 50 min, with continuous rotation on a 3D rotor to facilitate dissociation. After digestion, mechanical dissociation was achieved by vigorous pipetting to further enhance the yield of single cells. The resulting cell suspension was passed through a series of strainers (100, 70, and 40 μm) to isolate single cells and small cell aggregates.

The cells were washed with Dulbecco’s phosphate-buffered saline (DPBS, Servicebio, G4200) for 5 min and centrifuged at 300g for 5 min. Red blood cells were lysed using red blood cell lysis buffer (Servicebio, G2015), followed by an additional DPBS wash and a final centrifugation at 300g for 5 min. Cell pallets were resuspended in Matrigel (Corning, 354234) at a concentration of 0.5 × 10^7^ cells/ml on ice for further use.

### Primary SkMO production

Both NEOs and DEOs were produced using the same cell density. For NEOs, organoid domes were generated manually. To prepare, 24-well plates were prewarmed in a 37 °C incubator for 10 min before pipetting. Primary cell-laden Matrigel was then pipetted into the wells to form 10-μl domes. The plates were incubated at 37 °C for 15 min to allow the Matrigel domes to solidify. After solidification, culture medium was gently added to each well.

For DEOs, we adapted a method from our previous work [31–33]. Briefly, cell-laden Matrigel in the aqueous phase was loaded into a 1-ml syringe (Yuekang) and injected into a PTFE microfluidic tubing (Woer, 24T, inner diameter = 0.56 mm, outer diameter = 1.06 mm) by an injection pump (lead fluid). The inert oil phase (3M, Novec 7000) was loaded into a 10-ml syringe (Yuekang) and injected into another microfluidic channel by another injection pump (lead fluid). The 2 channels converged at a T-junction (homemade), where the aqueous and oil phases were injected at flow rates of 15 and 75 μl/min, respectively. This process resulted in the uniform division of Matrigel into cell-laden droplets, which were stored in long PTFE output tubing in queue. The whole process of droplet generation was carried out in a 4 °C fridge. After 15 min of solidification at 37 °C, the organoid precursors were printed into culture plates for subsequent culture, referred to as DEOs.

### Organoid culture

Both NEO and DEO cultures followed a proliferation–differentiation process. From D0 to D6, NEOs and DEOs were cultured in a primary SkMO proliferation medium (Meatoid, M0101), with medium changes every 2 d. On D6, the culture medium was switched to a primary SkMO differentiation medium (Meatoid, M0201), and the organoids were allowed to differentiate for 2 d to develop into mature SkMOs. The SkMOs could be maintained in the differentiation medium for extended culture as required.

### Electrical stimulation

Electrical stimulation was conducted using an ES plate (Meatoid, H0101), a pulse generator, and connective wires. The ES plate was sterilized with ultraviolet radiation for 2 h prior to use. At D8, the culture plate lid was replaced with the ES plate lid. Alternatively, SkMOs could be seeded directly into the ES plate at D0, allowing for continuous electrical stimulation throughout the culture period.

Each well of the ES plate contained a pair of copper–graphite electrode complexes at the left and right boundaries, where the graphite component was in direct contact with the differentiation medium. The inert nature of the graphite ensured the stability of the medium composition, minimizing interference from the electrodes and the electrical stimulation process. The copper components of the electrode complex pair were connected to the positive and negative outputs of a pulse generator. Independent electrical stimulation for each well could be achieved using a multichannel signal generator. Additionally, the electrode assemblies were connected as needed to enable parallel experiments with repeats. The electrodes were designed to have a minimal height, preventing interference with real-time monitoring via an inverted microscope (Nikon). Electrical stimulation was applied at D8 using a 10-V square wave with a 5% duty cycle.

### Contraction quantification

The contraction of SkMOs was recorded using the camera of an inverted microscope (Nikon) at 24 frames per second. Quantification of the contraction data was performed using the well-established MUSCLEMOTION algorithm [24,42,43]. In brief, the recorded bright-field WMV files were converted to uncompressed AVI files using the FFmpeg software. These AVI files were then processed with the MUSCLEMOTION macro in the ImageJ software. The resulting contraction data were normalized and further analyzed using Python 3 scripts.

### RT-qPCR

Total RNA was extracted using a commercial RNA extraction kit (Promega, LS1040) following the manufacturer’s instructions. Primary skeletal mouse tissues were manually homogenized on ice using a glass tissue homogenizer prior to lysis, while NEO and DEO samples were directly lysed by adding lysis buffer. The RNA concentration and purity were measured using a Nanodrop spectrophotometer (Thermo Fisher, ND-2000). Complementary DNA (cDNA) synthesis was performed using a reverse transcription kit (Takara, RR047A). RT-qPCR was then carried out using a qPCR kit (Promega, A6002) on a Bio-Rad CFX96 real-time PCR system, with specific primers listed in Table S3. Gene expression was quantified using the ΔΔCt method, normalized to β-actin gene expression as the reference.

### RNA-seq analysis

RNA-seq was performed on the Illumina PE150 sequencing platform by Chi Biotech (China). Skeletal muscle tissues were snap-frozen in liquid nitrogen immediately after collection. For NEO and DEO samples, tissues were immersed in TRIzol solution (15596018CN) and vigorously pipetted until no visible tissue fragments remained. Briefly, RNA extraction from tissues and cells was performed with stringent quality control, with RNA concentration and purity assessed using a NanoDrop spectrophotometer and RNA integrity evaluated on Agilent 4200 TapeStation. mRNA was enriched using oligo(dT) magnetic beads to target poly(A) tails. The enriched mRNA was fragmented and used as a template for first-strand cDNA synthesis with random oligonucleotides. Second-strand cDNA synthesis followed, using deoxynucleotide triphosphates and a second-strand enzyme. The resulting double-stranded cDNA underwent end repair, A-tailing, and adaptor ligation. Fragments approximately 200 to 300 bp in size were selected, followed by PCR amplification and purification, which completed the library preparation. The final libraries were quantified using Qubit 2.0 Fluorometer and verified for insert size with Agilent 4200 TapeStation. Sequencing was then performed on an Illumina platform using paired-end 150-bp sequencing (PE150). RNA-seq analysis was conducted using the Dr. Tom RNA-seq analysis platform (BGI) and Python scripts.

### Embedding and cryosectioning

Freshly isolated skeletal muscle tissue was washed 3 times with ice-cold DPBS. The tissue was then embedded in Tissue-Tek O.C.T. Compound (Sakura, 4853) using a custom-made polystyrene mold. To control the freezing speed and prevent cracks in the muscle, the mold was immersed in a metal cup filled with isopentane, which was then submerged in liquid nitrogen. NEO and DEO samples were collected at D8 and washed 3 times with ice-cold DPBS. After embedding in Tissue-Tek O.C.T. Compound, the samples were snap-frozen using liquid nitrogen. All tissues were cryosectioned into 8-μm-thick slices using a cryostat (Thermo Fisher, NX50) and mounted onto positively charged adhesive microscope slides (CITOTEST, 188105).

### Immunofluorescence

Cryosectioned slides were air-dried at room temperature (RT) for 15 min to prevent detachment. The slides were then washed 3 times with DPBS, each wash lasting 5 min. Permeabilization and blocking were carried out with a 1% bovine serum albumin solution (Sigma-Aldrich, A7888) containing 0.1% Triton X-100 (Sigma-Aldrich, X100) at RT for 1 h, followed by another set of three 5-min washes with DPBS. To reduce autofluorescence, the slides were treated with the TrueBlack lipofuscin autofluorescence quencher (Biotium, 23007) for 1 min, after which they were washed again with DPBS 3 times, for 5 min per wash. The slides were then incubated with primary antibodies (Table S4) at 4 °C overnight. The following day, the slides were washed 3 times with DPBS, each for 5 min, and then stained with secondary antibodies for 1 h at RT. After staining, another set of three 5-min washes with DPBS was performed. Finally, the slides were counterstained with 4′,6-diamidino-2-phenylindole (Sigma-Aldrich, D9542) at 1 μg/ml for 10 min at RT, followed by a final 5-min DPBS wash. The slides were mounted with one drop of glycerol (Sigma-Aldrich, G5516) and sealed with a coverslip using nail polish. Prepared slides were stored at −20 °C and imaged using a confocal microscope (Olympus, FV3000).

### Fluorescence-activated cell sorting

For the initial cell pool, since we performed sequential cell straining prior to organoid establishment, the initial pool already consisted of mononuclear cells. For NEOs and DEOs, organoids were digested with collagenase I for 30 min, followed by filtration through a 70-μm strainer. For DEO-emigrated cells, digestion was performed with 0.25% trypsin for 3 min, neutralized with serum-containing medium, and filtered through a 70-μm strainer. Here, we used mononuclear cells only in this analysis because mature myofibers contain multiple nuclei and are considerably larger, which may cause clogging in the flow cytometer.

Cells were first washed with DPBS and then resuspended in cell staining buffer (BioLegend). They were subsequently counterstained with CD31-APC750, CD45-FITC, CD106-PC7, Sca-1-BV510, CD140a/Pdgfra-BV421, CD140b/Pdgfrb-APC, and 7-AAD (all from BioLegend) for 20 min at RT, following the manufacturer’s recommended concentrations. After staining, cells were washed again with cell staining buffer before analysis using a flow cytometer (CytoFLEX, Beckman Coulter).

### ATP quantification

Cellular ATP levels were determined using CellTiter-Glo 2.0 Luminescent Assay (Promega) as per the manufacturer’s protocol. Organoids in black-walled 96-well plates were exposed to experimental compounds. Subsequently, 100 μl of ATP-detection reagent was added to each well, and plates were agitated gently for 10 min at RT. Following organoid lysis, luminescence signals reflecting ATP content were recorded using a microplate reader. Data were normalized to untreated controls.

### Cytotoxicity assessment via LDH release

LDH activity in culture supernatants was quantified to evaluate membrane integrity (Beyotime). Aliquots (120 μl) of supernatant were combined with 60 μl of the LDH detection reagent and incubated for 30 min at ambient temperature under light-protected conditions. Absorbance was measured at 490 nm, with 600 nm serving as the reference wavelength.

### Autophagosome detection with MDC staining

Autophagic vacuoles were labeled using MDC (Solarbio) according to standardized protocols. Cells were rinsed with buffer, incubated with MDC solution for 30 min at RT, and washed twice (3 min each). Fluorescent puncta were visualized using an inverted fluorescence microscope.

### 2-NBDG-based glucose uptake analysis

To model HG, SkMOs were cultured in 30 mM glucose (HG), while Ctrl received 17.5 mM glucose. After 48 h, SkMOs were transferred to glucose-free medium for 1 h to induce starvation. Glucose uptake was assayed by incubating samples for 30 min in glucose-free medium containing 75 μM 2-NBDG (MCE) and 10 nM insulin. Following DPBS washes, intracellular fluorescence was measured postlysis using a plate reader.

### Ach stimulation

Ach chloride (HY-B0282, MCE) was sequentially added to the SkMO culture medium, increasing the Ach concentration by 1 μM with each addition. Real-time SkMO contraction was monitored using a microscope (Nikon).

## Data Availability

The data supporting the results of this study can be obtained from the corresponding author upon reasonable request.

## Supplementary Materials

Supplementary 1 Figs. S1 to S5 Tables S1 to S4 Movies S1 to S7 References
