# Supplementary material for: Microdroplet-Engineered Skeletal Muscle Organoids from Primary Tissue Recapitulate Parental Physiology with High Reproducibility
Source: Research (Wash D C). 2025 May 15;8:0699. doi: 10.34133/research.0699 (PMC12078942; doi:10.34133/research.0699)
Supplement: Supplementary 1 — Figs. S1 to S5 Tables S1 to S4 Movies S1 to S7 References [file research.0699.f1.zip › SkMO_SI-20250112-CleanVersion.docx]

**Supporting Information for**

Microdroplet engineered skeletal muscle organoids from primary tissue recapitulate parental physiology with high reproducibility.

Jiawei Li, Yiming Yang, Ziqi Yi, Yu Zhu, Haowei Yang, Baiming Chen, Peter E Lobie, Shaohua Ma^*^.

*Shaohua Ma.

**Email:**  [ma.shaohua@sz.tsinghua.edu.cn](mailto:ma.shaohua@sz.tsinghua.edu.cn)

**This PDF file includes:**

Figures S1 to S5

Tables S1 to S4

Legends for Movies S1 to S7

SI References

**Other supporting materials for this manuscript include the following:**

Movies S1 to S7

Fig. S1. Immunofluorescence spatial consistency of NEO and DEO. (A) Schematic diagram illustrating the spatial analysis workflow. The fluorescence channel of the target marker was isolated, and the images were divided into multiple sub-images of equal size. Each sub-image was then thresholded into a binary format, and the ratio of positive area to total sub-image area was quantified for subsequent analysis. (B) Immunofluorescence images of Myh7 and MF20 in NEO and DEO, counterstained with DAPI. Furthermore, the MF20 images were subjected to an additional counterstain with Laminin. Scale bars, 100 µm. (C) Heatmap of sub-image expression. Statistical analysis was performed using a t-test, with statistical significance denoted as follows: * for p < 0.05, ** for p < 0.01, and *** for p < 0.001.

*
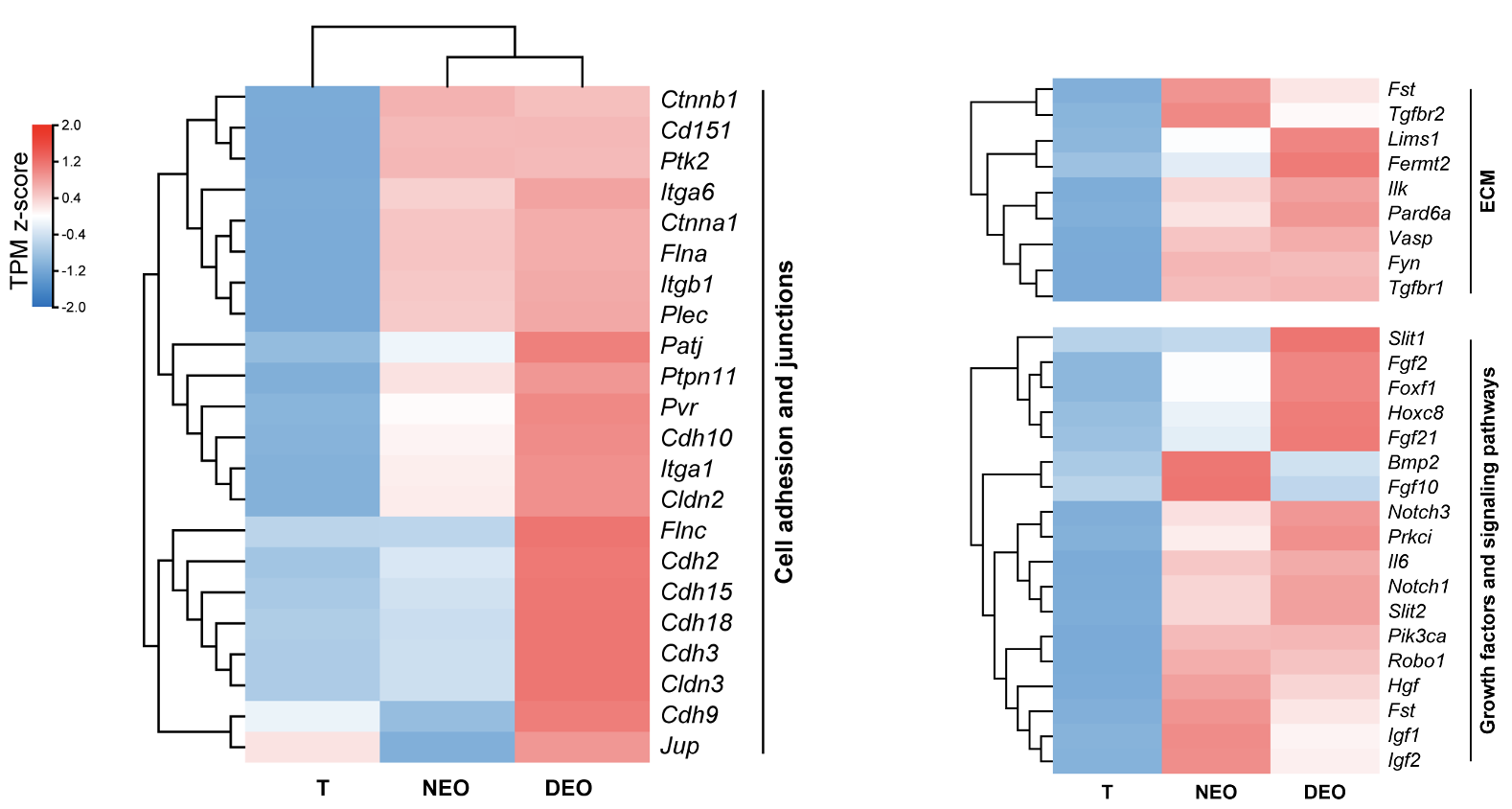
*

Figure S2. Heatmap of cell-cell communication genes in T, NEO and DEO.


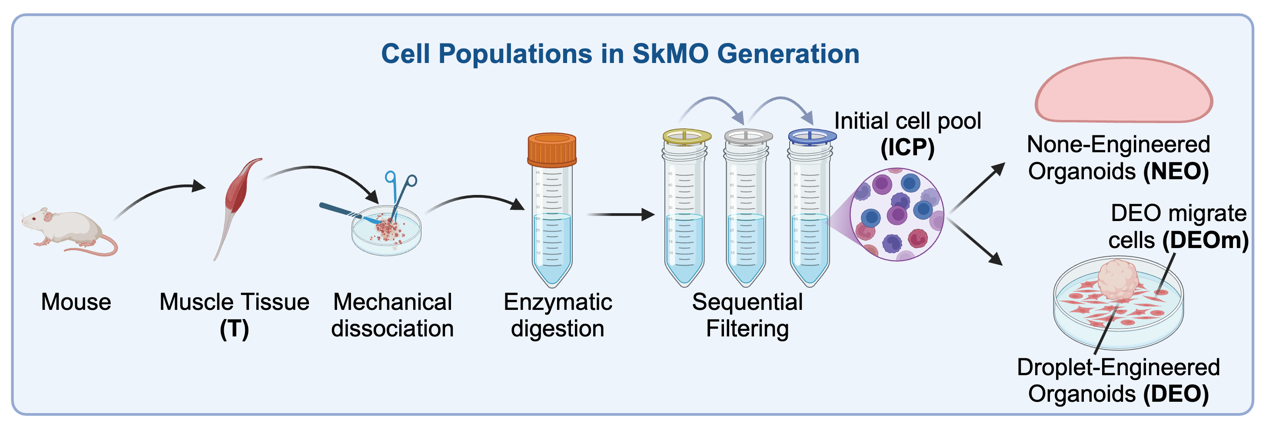


Figure S3. Cell populations in SkMO generation.

T, skeletal muscle tissue. ICP, initial cell pool after dissociation, digestion, filtering and erythrocyte lysis. NEO, Non-Engineered Organoids. DEO, Droplet-Engineered Organoids. DEOm, DEO migrated cells.


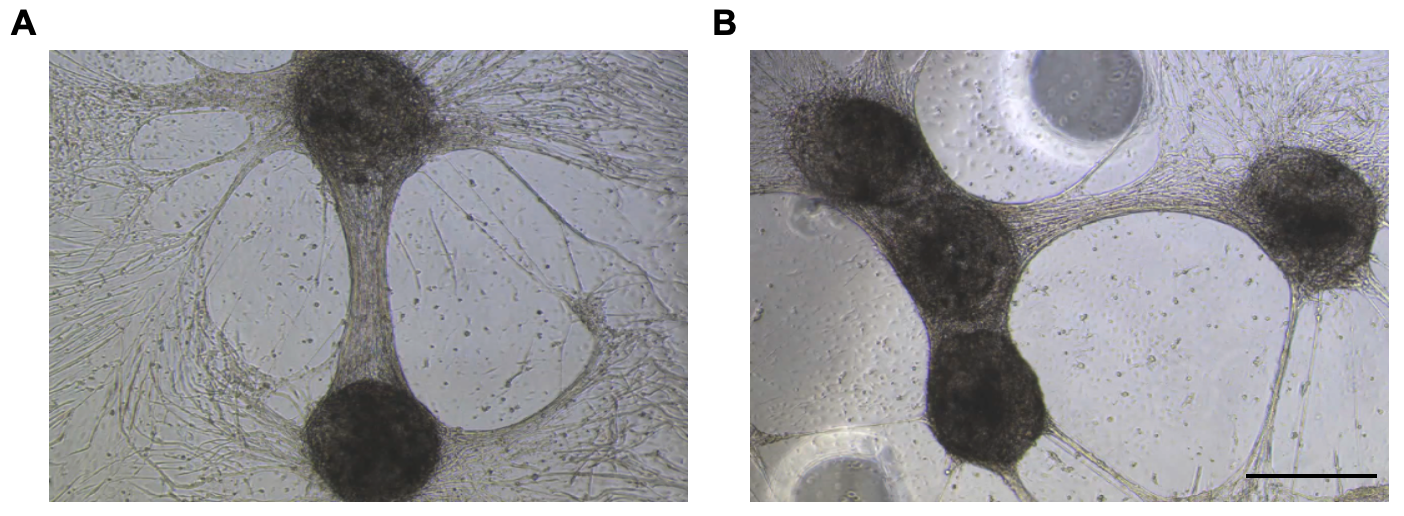


Fig. S4. Sparse assembly of DEOs. (A) Dual DEO assembly. Cells migrated from the DEOs and formed inter-DEO contractile connection muscle fibers. (B) Assembly of 4 DEOs. Scale bar, 500 µm.

Fig. S5. Electrical stimulation system and electrical stimulation parameter setup. (A) Schematic diagram of the Electrical Stimulation Plate (ES Plate). (B) Electrical stimulation system, showing the electrode complex connected to the signal generator. (C) Normalized contraction amplitude of DEO under 1 Hz electrical stimulation at various voltages (5V, 10V, 15V, 20V). (D) Normalized peak amplitude values from (C) across different stimulation voltages (5V, 10V, 15V, 20V). (E) Contraction amplitude of DEO under 1 Hz, 10V electrical stimulation with varying duty cycles (5%, 10%, 15%, 20%, 25%). (F) Peak morphology corresponding to the contraction in (E).

Table S1. Comparison of current SkMO research.

| Author | Shahriyari *et al.* ^1,2^ | Mavrommatis *et al.* ^3^ | Price *et al.* ^4^ | This work |
| --- | --- | --- | --- | --- |
| Cell source | hPSCs | hiPSCs | Mouse primary satellite cell, human myoblasts | Mouse primary muscle tissue |
| Sarcomere | Yes | Yes | No | Yes |
| Spontaneous Contraction | Yes | Yes | No | Yes |
| Stimulated contraction and frequency response | Yes | No | No | Yes |
| Fiber type validation | Gene, WB | Gene expression (Week 16) | No | Gene, qPCR, IF |
| 2D expansion estimated duration | >10 days | 10 days | 5-10 passages, 20-30 days | N&A |
| Organoid development period | 50 days | >60 days | 30 days | 8 days |
| Estimated total duration | >60 days | >70 days | 50-60 days | 8 days |
| Consistency | Low (tissue engineering) | Moderate (EB formation, Matrigel embedding) | Moderate (cell spontaneous aggregating) | High (Cascade-tubing microfluidics) |
| Throughput | 10s | 10-100 | ~100 | 500-1000s |

WB, Western Blotting. IF, Immunofluorescence. EB, Embryonic Body.

Table S2. FACS gating of skeletal muscle cells

| **Cell Type** | **Gating** |
| --- | --- |
| Immune cell | CD45+ |
| Endothelial | CD45-/CD31+ |
| FAP | CD45-/CD31-/Vcam-/Sca-1+ |
| MP | CD45-/CD31-/Vcam+/Sca-1- |
| Fibroblast | CD45-/CD31-/Vcam-/Sca-1-/Pdgfra+ |
| Pericyte | CD45-/CD31-/Pdgfrb+ |

Table S3. RT-qPCR primer sequences.

| **Gene** | **Sequence (5’ to 3’)** |
| --- | --- |
| *β-Actin* | Fw - CATTGCTGACAGGATGCAGAAGG |
|  | Rv - TGCTGGAAGGTGGACAGTGAGG |
| *Pax7* | Fw - GTTCGGGAAGAAAGAGGACGAC |
|  | Rv - GGTTCTGATTCCACATCTGAGCC |
| *Myod1* | Fw - GCACTACAGTGGCGACTCAGAT |
|  | Rv - TAGTAGGCGGTGTCGTAGCCAT |
| *Myog* | Fw - CCATCCAGTACATTGAGCGCCT |
|  | Rv - CTGTGGGAGTTGCATTCACTGG |
| *Myh2* | Fw - GCGACTTGAAGTTAGCCCAGGA |
|  | Rv - CTCGTCCTCAATCTTGCTCTGC |
| *Myh4* | Fw - AGAGCCAAGAGGAAACTGGAGG |
|  | Rv - CTCGTCCTCAATCTTGCTCTGC |
| *Myh7* | Fw - GCTGGAAGATGAGTGCTCAGAG |
|  | Rv - TCCAAACCAGCCATCTCCTCTG |

Table S4. Antibody list.

| **Antibody** | **Host** | **Dilution** | **Source** | **Catlog** |
| --- | --- | --- | --- | --- |
| Pax7 | Mouse | 1:10 | DSHB | AB_528428 |
| Myod | Rabbit | 1:100 | Proteintech | 18943-1-AP |
| MF20 | Mouse | 1:10 | DSHB | AB_2147781 |
| Myh2 | Mouse | 1:50 | DSHB | AB_2147165 |
| Myh4 | Mouse | 1:100 | DSHB | AB_2266724 |
| Myh7 | Rabbit | 1:500 | Servicebio | GB111857 |
| Rabbit 488 | Goat | 1:500 | Abcam | ab150077 |
| Mouse 488 | Goat | 1:500 | Abcam | ab150113 |
| Rabbit 594 | Goat | 1:500 | Abcam | ab150080 |

Movie S1. **NEO spontaneous contraction**.

Movie S2. **DEO spontaneous contraction**.

Movie S3. **DEO migrated cell contraction.**

Movie S4. **NEO contraction under 1 Hz electrical stimulation**.

Movie S5. **DEO contraction under 1 Hz electrical stimulation**.

Movie S6. **DEO tetanic contraction.**

Movie S7. **Ach Stimulation contraction.**

**SI References**

1. Shahriyari, M., Rinn, M., Hofemeier, A.D., Babych, A., Zimmermann, W.H., and Tiburcy, M. (2024). Protocol to develop force-generating human skeletal muscle organoids. STAR Protoc *5*, 102794. 10.1016/j.xpro.2023.102794.

2. Shahriyari, M., Islam, M.R., Sakib, S.M., Rinn, M., Rika, A., Krüger, D., Kaurani, L., Gisa, V., Winterhoff, M., Anandakumar, H., et al. (2022). Engineered skeletal muscle recapitulates human muscle development, regeneration and dystrophy. J Cachexia Sarcopenia Muscle *13*, 3106-3121. 10.1002/jcsm.13094.

3. Mavrommatis, L., Jeong, H.-W., Kindler, U., Gomez-Giro, G., Kienitz, M.-C., Stehling, M., Psathaki, O.E., Zeuschner, D., Bixel, M.G., Han, D., et al. (2023). Human skeletal muscle organoids model fetal myogenesis and sustain uncommitted PAX7 myogenic progenitors. eLife *12*, RP87081. 10.7554/eLife.87081.

4. Price, F.D., Matyas, M.N., Gehrke, A.R., Chen, W., Wolin, E.A., Holton, K.M., Gibbs, R.M., Lee, A., Singu, P.S., Sakakeeny, J.S., et al. (2024). Organoid culture promotes dedifferentiation of mouse myoblasts into stem cells capable of complete muscle regeneration. Nature Biotechnology. 10.1038/s41587-024-02344-7.
